# Metrorrhagia in Interstitial Nephritis

**Published:** 1905-06

**Authors:** Charles Greene Cumston

**Affiliations:** Boston, Mass.


### Metrorrhagia in Interstitial Nephritis.

By CHARLES GREENE CUMSTON, M. D., Boston, Mass.

DISEASES of the circulatory apparatus may give rise to metrorrhagia, whether this may be due to passive congestion during the course of mitral disease, or in dilatation of the right ventricle in pulmonary emphysema; or, on the other hand, it may be due to active congestion during the progress of interstitial nephritis, in which high arterial tension and vascular changes, which are frequently concomitant, predispose to rupture of the vessels. Hepatic cirrhosis giving rise to passive congestion, may produce metrorrhagia, occasionally following a stasis produced by some external pressure, such as tightly laced corsets, and the like. There are still other causes in play, such, for instance, as a stasis resulting from compression of the vena cava by an abdominal tumor, or a fluxion following the repeated use of strong purgatives or emmenagogues. After ligature of the umbilical cord one occasionally sees a slight metrorrhagia in a newly born infant, which is due to a venous stasis resulting from the ligature during the first forty-eight hours following birth. The variations in the general conditions of the circulation also explain the metrorrhagia which has been met with in females who have left the low lands to take up their abode in high altitudes.

Interstitial nephritis occupies quite an important place in disturbances of the circulation and which result in loss of blood from the uterus. Generally speaking, the hemorrhage under these circumstances is of slight amount and only temporary, but there are many exceptions to this rule, and Violet has recently reported three cases where the amount of blood lost was sufficient to produce a marked anemia in the patients. In one case, that of a woman forty-two years of age, who had a miscarriage, for which she was curetted three years previously, the flow of blood after this was continuous and accompanied by clots, which reduced her to a condition of extreme weakness. Ergotin was several times given, but had no influence over the flow. Examination showed a slightly granulous cervix, but the uterus was normal, both in size and position. No evidence of a fibroid tumor could be detected. On the other hand, there was a marked arterial hypertension with a tendency to a cardiac galop and the urine, which was very limpid, contained a marked trace of albumin. In the other two cases, the same condition of arterial hypertension and albuminuria were present, while the uterus and the adnexa were found normal. In both these cases curetment only brought away normal uterine mucosa.

It is of utmost importance for the practitioner to be aware of this cause of metrorrhagia. Ergotin is one of the most common remedies for uterine hemorrhage and this drug is an energetic vasoconstrictor and hypertensor; consequently, when given under these conditions, it becomes, so to speak, an accomplice to the disease which in itself makes itself known by a marked arterial hypertension. Should one prescribe ergot to a patient the subject of interstitial nephritis, it simply means that the loss of blood will be prolonged. Those patients should in reality be placed upon a general treatment which will produce hypotension, such as a milk and vegetable diet consisting of eggs, green vegetables, fruit and not more than a pint of liquid in twenty-four hours. To this diet theobromin and similar drugs may be added. If this regime is not sufficient a local treatment consisting of hot vaginal irrigations, or destruction of the uterine mucosa by the curet, must be resorted to.

The conclusion of all this is that which dictates the daily conduct of the physician—namely, before prescribing the treatment the patient should be carefully examined and the causative factor should be determined.

Interstitial nephritis is without any doubt quite a rare cause of metrorrhagia and, in a female during the genital period, one would naturally first think of a fibroid or a retention of the placenta. If the subject is more advanced in age one should not adopt the vague and meaningless term of metrorrhagia of the menopause, without, in the first place, making certain that a carcinoma of the uterine body is not developing. It is quite impossible to make a diagnosis without the sense of sight and especially of touch in these cases and, for this reason, intrauterine exploration is indicated. As an example of the importance of this may be mentioned a case, that of a young virgin, nineteen years of age, in whom a mucous polypus, the size of a large bean, was found within the uterine cavity. The patient entered the hospital for extremely abundant hemorrhage, which had been present for over a year and, on examination bimanually, the uterus was not enlarged, while the cervix was perfectly hard and normal and the real condition of affairs was not ascertained until an intrauterine examination had been made.

In cases of retention of the placenta the dilatation of the cervix, with colicky pains during a metrorrhagia, indicates that there is some foreign body within the uterus which the organ is endeavoring to expel. These symptoms, however, are not always constant and a retention of the placenta may escape detection by the usual methods of exploration, just as is the case for interstitial fibroids of small size which project into the uterine cavity, or certain malignant neoplasms at their beginning.

Under such circumstances an intrauterine examination should never be neglected and, if carried out with all the rules of asepsis, no harm will result. The exploring finger will immediately appreciate the condition of the mucosa and, if necessary, curettage may be done in order to make an histological examination.

Other causes of metrorrhagia may be eliminated, such, for example, as that accompanying fungous endometritis, salpingo-ovaritis, infectious diseases, the commencement of tuberculosis, diabetes and similar affections.

871 Beacon Street.

